# Assessing fatigue in women over 50 years with rheumatoid arthritis: a comprehensive case-control study using the FACIT-F scale

**DOI:** 10.3389/fmed.2024.1418995

**Published:** 2024-07-25

**Authors:** Lidia Valencia-Muntalà, Carmen Gómez-Vaquero, Laura Berbel-Arcobé, Diego Benavent, Paola Vidal-Montal, Xavier Juanola, Javier Narváez, Joan M. Nolla

**Affiliations:** Department of Rheumatology, IDIBELL-Hospital Universitari de Bellvitge, University of Barcelona, Barcelona, Spain

**Keywords:** fatigue, rheumatoid arthritis, FACIT-F scale, comorbidity, case-control study

## Abstract

**Introduction:**

Data on prevalence of fatigue in rheumatoid arthritis (RA) patients in the era of biological treatments remains scarce, with a lack of case-control studies. This study evaluates the prevalence of fatigue in Spanish women over 50 years with RA using the Functional Assessment of Chronic Illness Therapy-Fatigue (FACIT-F) scale, explores its association with RA-related variables, and seeks to identify the primary factors influencing fatigue. Ultimately, our objective is to underscore the clinical significance of fatigue as a comorbidity and to advocate for its systematic evaluation in routine clinical practice.

**Methods:**

In a case-control study at a tertiary university hospital, 191 women over 50 years (mean age: 67.5 ± 8.8 years) meeting ACR 2010 criteria for RA and age-matched controls were assessed using the FACIT-F scale, SF-12 questionnaire, and RA-related clinical measures.

**Results:**

Fatigue was significantly more prevalent in the RA group (61%) compared to controls (37%, *p* < 0.001), with RA patients showing lower mean FACIT-F scores (36.0 ± 10.6 vs. 40.0 ± 0.6, *p* < 0.001). Correlations were noted between FACIT-F scores and C-reactive protein, DAS28, RAPID3, HAQ, and SF-12 scores. A multivariate analysis was performed and four models generated. The final model, with an R^2^ of 0.817, indicates that fatigue is significantly influenced by disease activity (RAPID 3) and mental and physical health (SF12) and age, explaining 81.7% of the variance in fatigue.

**Conclusion:**

Fatigue remains significantly prevalent and severe in women over 50 years with RA, strongly linked to disease activity, disability, and diminished quality of life. Systematic fatigue assessment and targeted strategies in clinical settings are essential to address this widespread issue. Future research should explore targeted interventions tailored to this demographic to enhance quality of care.

## Introduction

Rheumatoid arthritis (RA), the most commonly diagnosed systemic autoimmune disease worldwide, manifests as a complex condition with persistent and progressive joint and systemic symptoms, one that significantly elevates the risk of disability and mortality (1). It spans a broad spectrum of manifestations that amplify its overall impact, highlighting the essential need for continuous, integral strategies for disease management.

Recent advances in the treatment of RA have markedly enhanced patient outcomes (2), leading to substantial reductions in disease activity and, in certain instances, achieving remission through the targeting of inflammatory pathways. Concurrently, these improvements in managing RA activity have brought greater awareness to associated comorbidities. Beyond conventional concerns like cardiovascular risks, osteoporosis, and infections, clinical practice (3) is increasingly acknowledging other significant issues, including anxiety, depression, sexual dysfunction, sarcopenia, and fatigue.

Fatigue is a complex phenomenon, multifactorial and multidimensional, lacking a universally accepted definition (4). It is experienced as an overwhelming sense of exhaustion that significantly diminishes an individual’s ability to perform daily activities (5). Recognized worldwide, fatigue can occur as an independent condition or in association with medical procedures and treatments. While it is recognized that patients with RA are more likely to experience fatigue (5–7), data on its prevalence in the era of biological treatments remains relatively sparse, with a lack of case-control studies.

The inherently subjective nature of fatigue, coupled with the challenge of quantifying it, has prompted the creation of various assessment tools (8). Despite these efforts, the establishment of clear guidelines for choosing appropriate instruments for research and clinical practice in rheumatic diseases has been challenging. A thorough review (9) was unable to pinpoint a single scale or instrument as the optimal means for measuring fatigue across different rheumatological conditions.

Common measures for assessing fatigue in RA (10) encompass numeric rating scales, such as the Visual Analogue Scale (VAS), and questionnaires, including, among others, the Bristol Rheumatoid Arthritis Fatigue Multidimensional Questionnaire (BRAF-MDQ), the SF-36 Vitality Domain, the Patient-Reported Outcomes Measurement Information System (PROMIS-29) Fatigue T-score, and the Functional Assessment of Chronic Illness Therapy-Fatigue (FACIT-F).

Among all, the FACIT-F scale (11), recognized for its detailed measurement of fatigue in individuals with chronic diseases (9), balances assessments of both physical and emotional well-being. Recently, it has been adopted as a patient-reported outcome in several RA clinical trials (12–14) of JAK inhibitors, emphasizing the value of its psychometric properties.

The exact causes of fatigue in RA are not fully understood (15, 16). Although traditionally associated with inflammation, the persistence of fatigue in many RA patients, despite advancements in anti-inflammatory therapies, underscores both its complexity the need for further investigation.

This study aimed to assess the prevalence of fatigue among a large cohort of elderly Spanish women with RA using the FACIT-F scale. Additionally, we explored the relationship between FACIT-F scores and critical RA-related variables to develop an understanding of the key determinants of this condition. Ultimately, our objective was to approach the clinical relevance of this comorbidity, evaluating the feasibility of incorporating systematic analysis of this condition into routine clinical practice.

## Materials and methods

### Study population

This observational case-control study focused on women over 50 with RA, comparing them to age-matched controls. RA patients were diagnosed based on the 2010 ACR criteria and recruited during routine rheumatology visits at a tertiary university hospital. Controls were sourced through three primary methods: accompanying individuals of patients at the rheumatology service, those with non-inflammatory musculoskeletal disorders (mainly soft tissue disorders), and individuals attending the hospital for conditions unrelated to musculoskeletal diseases. We carefully ensured that neither the RA patients nor the control group had any conditions known to cause fatigue, such as cancer, heart or respiratory failure, chronic liver or kidney diseases, or central sensitivity syndromes like fibromyalgia. All participants gave written informed consent, and the local ethics committee approved the study (reference: PR057/20).

### Study variables

#### Sociodemographic and anthropometric data

• Age
• Body mass index (BMI): BMI is the ratio of human body weight to squared height expressed in kg/m^2^. It has been categorized as follows: < 18.5 kg/m^2^ is considered underweight; from 18.5 to 25 kg/m^2^, normal range; from 25 to 30 mg/m^2^, overweight; and > 30 kg/m^2^, obese.
• Tobacco use: We categorized the patient population into three groups based on tobacco use: never smokers, current smokers, and former smokers.
• Physical activity: We categorized the patient population based on their levels of physical activity into four groups: none, sporadic, regular with low intensity, and regular with high intensity.

#### Fatigue assessment

The FACIT-F scale (11) was employed to measure fatigue levels. This scale includes items rated on a scale from 0 to 4, yielding a total possible score that ranges from 0 to 52, where lower scores signify greater levels of fatigue. While there is no universally accepted cutoff for the presence of fatigue, for the purposes of our study, we pre-established a score below 40 to denote “fatigue” which is in line with the several studies available in the literature (17, 18). Additionally, we have indicated the percentage of patients with a FACIT-F score of < 30, which some authors (19) consider indicative of “significant fatigue.”

#### Evaluation of health-related quality-of-life

We employed the SF-12 questionnaire (20). The SF-12, or Short Form Health Survey, is a 12-item questionnaire designed to measure health-related quality of life. It assesses functional health and well-being from the patient’s perspective. The SF-12 includes two composite scores representing physical and mental health. It is a condensed version of the SF-36 survey, aimed at reducing the burden on respondents while preserving essential health status information. For each participant, two summary scores were calculated: one for physical health and another for mental health. The scores range from 0 to 100, where a higher value indicates a better health-related quality of life.

### RA assessment

• Evaluation of RA history: (a) disease duration; (b) positivity of rheumatoid factor (RF), along with their titers; (c) positivity for anti-citrullinated peptide antibodies (ACPA), along with their titers; (d) current treatment (glucocorticoids, conventional disease-modifying antirheumatic drugs, biological disease-modifying antirheumatic drugs, Jak inhibitors).
• Analytical evaluation. We considered the following parameters: (a) albumin levels; (b) erythrocyte sedimentation rate (ESR); (c) C-reactive protein (CRP); and (d) hemoglobin levels. The values corresponding to the last analytical study carried out were considered.
• Evaluation of RA activity using metrological indices. We utilized two indices for this purpose: (a) the Disease Activity Score 28 (DAS28) and (b) the Routine Assessment of Patient Index Data 3 (RAPID3).
  (a) DAS28 (21) is a composite index of disease activity comprising tender and swollen joint counts in 28 joints, the Patient’s Global Assessment of Disease Activity and the ESR. The higher the score, the higher the activity level. A value < 2.6 suggests disease remission, a value between ≥ 2.6 and ≤ 3.2 suggests low disease activity, a value > 3.2− ≤ 5.1 suggests moderate disease activity and, finally, a value > 5.1 suggests high disease activity.
  (b) RAPID3 (22) is a validated index for measuring disease activity in patients with RA that includes three measures self-reported by the patient: pain, physical function, and global assessment of the disease. The higher the score, the higher the activity level. A value ≤ 3 suggests disease remission, a value between 3.01 and 6 suggests low disease activity, a value between 6.01 and 12 suggests moderate disease activity and a value > 12 suggests high disease activity.
• Evaluation of disability. We used the Health Assessment Questionnaire (HAQ) (23). This questionnaire assesses physical functioning as difficulty performing daily living activities; the score ranges from 0 to 3. The higher the score, the higher the disability level.

### Statistical analysis

To determine the sample size necessary for our case-control study, we utilized the following parameters: a prevalence of fatigue estimated at 50% among cases and 35% among controls, a significance level (alpha) set at 0.05, and a power (1−beta) of 0.8, reflecting an 80% power to detect a significant difference. The ratio of cases to controls was established at 1:1.

The calculations indicated that a sample size of 169 cases and 169 controls, totaling 338 participants, was required to detect a significant difference in fatigue prevalence between the case group (women with RA) and the control group, at the specified significance and power levels.

Data are presented as the mean plus or minus the standard deviation/median and interquartile range for continuous variables and as a number and percentage for categorical variables. Prevalence rates are given as percentages. Continuous variables were tested for normality using the Kolmogorov-Smirnov test. Differences among parametric variables were assessed using ANOVA; for non-parametric variables, we used the U-Mann-Whitney or Kruskal-Wallis tests, when indicated. Differences among categorical variables were evaluated by the chi-squared test.

To assess the relationship between the variables of interest in this study, a correlation analysis using Pearson’s correlation coefficient was conducted. This analysis allowed us to examine linear associations between pairs of variables and determine the strength and direction of these relationships.

A multivariate study by multiple regression including all the variables that correlated with FACT-F plus age, BMI and RA disease duration was used to identify independent factors influencing fatigue.

## Results

Table 1 presents the characteristics of the 191 patients with RA included in the study. In the cohort the average age was 67.5 ± 8.8 years, with a mean BMI of 27.9 ± 5.4 kg/m^2^. The average disease duration among the participants was 16.8 ± 10.3 years. DAS28 scores showed 40.5% in remission and 32% with moderate disease activity, while RAPID3 scores revealed 34% in moderate and another 34% in high disease activity. The HAQ score suggested low disability, and 90% were on DMARDs. FACIT- F and SF-12 scores indicated significant fatigue and reduced quality of life, highlighting the substantial impact of RA on patient well-being.

**TABLE 1:** Characteristics of the patients with RA (n: 191) included in the study.

Age (years)	67.5 ± 8.8
BMI (kg/m^2^)	27.9 ± 5.4
Underweight (*n*, %)	0
Normal range (*n*, %)	64 (33.5%)
Overweight (*n*, %)	72 (37.5%)
Obese (*n*, %)	55 (29%)
**Smoking (n: 188)**	
Never	164 (87.2%)
Former	2 (1.1%)
Current	22 (11.7%)
**Physical activity (n: 187)**	
None	94 (50.3%)
Sporadic	36 (19.3%)
Regular with low intensity	55 (29.4%)
Regular with high intensity	2 (1.1%)
Albumin (g/L)	44,0 ± 3,4
Albumin < 35 g/L (*n*, %)	4 (2.1%)
Disease duration (years)	16.8 ± 10.3
RF + (n: 171) (*n*, %)	122 (71%)
RF titer (only RF+) (UI/L)	208 ± 415
ACPA + (*n*: 172) (*n*, %)	122 (71%)
ACPA titer (only ACPA+) (U/L)	369 ± 672
ESR (mm/h)	24.8 ± 20.8
CRP (mg/dL)	5.2 ± 6.1
Hemoglobin (g/dL)	13.3 ± 1.2
DAS28	2.9 ± 1.1
Remission (*n*, %)	77 (40.5%)
LDA (*n*, %)	47 (24.5%)
MDA (*n*, %)	61 (32%)
HDA (*n*, %)	6 (3%)
RAPID3 (*n*:161)	9.7 ± 6.9
Remission (*n*, %)	38 (24%)
LDA (*n*, %)	13 (8%)
MDA (*n*, %)	55 (34%)
HDA (*n*, %)	55 (34%)
HAQ	0.16 ± 0.80
**Current medication**	
Glucocorticoids (*n*, %)	89 (46.5%)
cDMARDs (*n*, %)	172 (90%)
bDMARDs (*n*, %)	68 (36%)
Jak inhibitors (*n*, %)	10 (5%)
**FACIT-F**	
Mean ± SD	36.0 ± 10.6
Median [IQ range]	38 [30; 43]
FACIT-F < 40 (*n*, %)	116 (61%)
FACIT-F < 30 (*n*, %)	47 (25%)
**SF-12**	
Mental health	45.1 ± 11.4
Physical health	36.8 ± 9.5

BMI, body mass index; RF, rheumatoid factor; ACPA, anti-citrullinated peptides antibodies; ESR, erythrocyte sedimentation rate; CRP, C-reactive protein; DAS28, Disease Activity Score 28; LDA, low disease activity; MDA, moderate disease activity; HDA, high disease activity; RAPID3, Routine Assessment of Patient Index Data 3; HAQ, Health Assessment Questionnaire; cDMARDs, conventional disease-modifying antirheumatic drugs; bDMARDs; biological disease-modifying antirheumatic drugs; FACIT-T, Functional Assessment of Chronic Illness Therapy-Fatigue; SD, standard deviation; IQ, interquartile; SF-12, Short Form Health Survey-12.

Table 2 shows a comparison between patients with RA and controls. The proportion of patients with fatigue (FACIT-F score < 40) was 61%, significantly higher (*p* < 0. 001) than observed in the control group (35%). In 47 patients (25%), fatigue was considered significant.

**TABLE 2:** Comparison of patients with RA and controls.

	Patients (*n*: 191)	Controls (*n*: 198)	*p*
Age (years)	67.5 ± 8.8	67.3 ± 9.2	ns
BMI (kg/m^2^)	27.9 ± 5.4	27.8 ± 5.3	ns
Underweight (*n*, %)	0	4 (2%)	ns
Normal range (*n*, %)	64 (34%)	57 (30%)	
Overweight (*n*, %)	72 (37%)	73 (38%)	
Obese (*n*, %)	55 (29%)	60 (30%)	
Hemoglobin (g/dL)	13.3 ± 1.2	13.7 ± 1.1	< 0.01
**FACIT-F**			
Mean ± SD	36.0 ± 10.6	40.0 ± 9.6	< 0.001
Median [IQ range]	38 [30; 43]	35 [42;47]	< 0.001
FACIT-F < 40 (*n*, %)	116 (61%)	74 (37%)	< 0.001
FACIT-F < 30 (*n*, %)	47 (25%)	30 (15%)	< 0.05
**SF-12**			
Mental health	45.1 ± 11.4	50.2 ± 10.1	< 0.001
Physical health	36.8 ± 9.5	44.0 ± 11.5	< 0.001

BMI, body mass index; FACIT-T, Functional Assessment of Chronic Illness Therapy-Fatigue; SD, standard deviation; IQ, interquartile; SF-12, Short Form Health Survey-12.

Mean value of FACIT-F in RA patients was 36.0 ± 10.6. This value was significantly lower (*p* < 0.001) than that obtained in the control group (40.0 ± 9.6).

Patients with RA presented a significantly lower values of hemoglobin levels. In addition, the scores of both dominions of SF-12 were significantly lower than in the control group.

Table 3 underline the profound impact of fatigue on disease burden. Patients with RA and fatigue displayed significantly higher disease activity and poorer quality of life compared to those without fatigue. While demographic and clinical characteristics such as age, BMI, disease duration and RF and ACPA positivity were similar across groups, the impact of fatigue was evident in higher disease activity scores and quality of life measurements.

**TABLE 3:** Characteristics of the patients with RA and the differences between the ones without and with fatigue.

	Without fatigue (n: 75)	With fatigue (n: 116)	p
Age (years)	67.6 ± 10.0	67.3 ± 8.1	ns
BMI (kg/m^2^)	27.2 ± 5.7	28.3 ± 5.2	ns
Underweight (*n*, %)	0 (−)	0 (−)	
Normal range (*n*, %)	31 (41%)	33 (28%)	
Overweight (*n*, %)	21 (28%)	51 (44%)	
Obese (*n*, %)	23 (31%)	32 (28%)	ns
**Smoking (n: 188)**			
Never	66 (90.4%)	98 (85.2%)	
Ever	1 (1.4%)	1 (0.9%)	
Current	6 (8.2%)	16 (13.9%)	ns
**Physical activity (n: 187)**			
None	33 (45.2%)	61 (53.5%)	
Sporadic	11 (15.1%)	25 (21.9%)	
Regular with low intensity	27 (37.0%)	28 (24.6%)	
Regular with high intensity	2 (2.7%)	0 (-)	ns
Albumin (g/L)	44,1 ± 3,4	44,0 ± 3,5	ns
Albumin < 35 g/L (*n*, %)	2 (2.7%)	2 (1.7%)	ns
Disease duration (years)	17.1 ± 11.2	16.6 ± 9.8	ns
RF + (*n*: 171) (*n*, %)	50 (73%)	72 (71%)	ns
RF titer (only RF+) (UI/L)	144 ± 220	266 ± 531	ns
ACPA + (*n*: 172) (*n*, %)	46 (69%)	76 (72%)	ns
ACPA titer (only ACPA+) (U/L)	348 ± 616	386 ± 718	ns
ESR (mm/h)	20.6 ± 18.2	27.4 ± 22.0	ns
CRP (mg/dL)	4.5 ± 4.0	5.7 ± 7.1	ns
Hemoglobin (g/dL)	13.4 ± 1.2	13.3 ± 1.2	ns
DAS28	2.5 ± 0.9	3.2 ± 1.1	< 0.001
Remission (*n*, %)	44 (58%)	33 (28%)	
LDA (*n*, %)	17 (23%)	30 (26%)	
MDA (*n*, %)	14 (19%)	47 (41%)	
HDA (*n*, %)	0 (−)	6 (5%)	< 0.001
RAPID3	4.6 ± 3.8	12.6 ± 6.6	< 0.001
Remission (*n*, %)	27 (46%)	11 (11%)	
LDA (*n*, %)	8 (14%)	5 (5%)	
MDA (*n*, %)	23 (39%)	32 (32%)	
HDA (*n*, %)	1 (1%)	54 (54%)	< 0.001
HAQ	0.08 ± 0.19	0.22 ± 0.34	< 0.01
**Current medication**			
Glucocorticoids (*n*, %)	30 (40%)	59 (51%)	ns
cDMARDs (*n*, %)	70 (93%)	102 (89%)	ns
bDMARDs (*n*, %)	24 (32%)	44 (38%)	ns
Jak inhibitors (*n*, %)	2 (3%)	8 (7%)	ns
**SF-12**			
Mental health	50.9 ± 9.5	41.5 ± 11.0	< 0.001
Physical health	43.5 ± 8.5	32.7 ± 7.5	< 0.001

BMI, body mass index; RF, rheumatoid factor; ACPA, anti-citrullinated peptides antibodies; ESR, erythrocyte sedimentation rate; CRP, C-reactive protein; DAS28, Disease Activity Score 28; LDA, low disease activity; MDA, moderate disease activity; HDA, high disease activity; RAPID3, Routine Assessment of Patient Index Data 3; HAQ, Health Assessment Questionnaire; cDMARDs, conventional disease-modifying antirheumatic drugs; bDMARDs, biological disease-modifying antirheumatic drugs; SF-12, Short Form Health Survey-12.

In RA patients, FACIT-F correlated significantly with clinical and quality of life parameters (Table 4). Negative correlations with CRP, DAS 28, RAPID3 and HAQ highlighted that increased inflammation, disease activity, higher disability and perceived disease severity were associated with greater fatigue. Positive correlations with SF-12 Mental Health and Physical Health demonstrated that lower fatigue levels are associated with better quality of life.

**TABLE 4:** Bivariate correlations between FACIT and the rest of variables.

r	FACIT-F	CRP	DAS28	RAPID3	HAQ	SF-12 MH	SF-12 PH
FACIT-F	–						
CRP	−0.20*	−					
DAS28	−0.38***	0.40***	−				
RAPID3	−0.72***	ns	0.47***	−			
HAQ	−0.33***	ns	0.27***	0.43***	−		
SF−12 MH	0.51***	ns	−0.25**	−0.37***	ns	−	
SF−12 PH	0.59***	ns	−0.40***	−0.62***	−0.30***	ns	−

FACIT-T, Functional Assessment of Chronic Illness Therapy-Fatigue; CRP, C-reactive protein; DAS28, Disease Activity Score 28; RAPID3, Routine Assessment of Patient Index Data 3; HAQ, Health Assessment Questionnaire; SF-12, Short Form Health Survey-12; MH, mental health; PH, physical health.

*p < 0.05,

**p < 0.01,

***p < 0.001.

A stepwise multiple regression was conducted to identify factors influencing fatigue measured by FACIT-F, incorporating variables correlated with FACT-F, age, BMI, and RA disease duration. Four models were generated. Model 1 included RAPID 3, showing a significant negative relationship with fatigue (B = −1.159, *p* < 0.001). Model 2 added SF12 mental showing a significant positive relationship (B = 0.286, *p* < 0.001). Model 3 included the physical component of SF12. Model 4 added age, with a smaller effect (B = 0.153, *p* = 0.011). The final model, with an *R*^2^ of 0.817, indicates that fatigue is significantly influenced by disease activity (RAPID 3) and mental and physical health (SF12) and age, explaining 81.7% of the variance in fatigue. The coefficients and significance of each variable in the final model are presented in Table 5.

**TABLE 5:** Multivariate analysis including all the variables that correlated with FACIT-F plus age, BMI and RA disease duration.

	Constant	Coefficient	*p*	*R* ^2^
**Model 1**				
RAPID3	46.418	−1.159	< 0.001	0.726
**Model 2**				
RAPID3	31.979	−0.989	< 0.001	0.775
SF12 MH		0.286	< 0.001	
**Model 3**				
RAPID3	14.885	−0.674	< 0.001	0.808
SF12 MH		0.322	< 0.001	
SF12 PH		0.341	< 0.001	
**Model 4**				
RAPID3	3.282	−0.651	< 0.001	0.817
SF12 MH		0.334	< 0.001	
SF12 PH		0.357	< 0.001	
Age		0.153	0.011	

RAPID3, Routine Assessment of Patient Index Data 3; SF-12, Short Form Health Survey-12; MH, mental health; PH, physical health.

## Discussion

Fatigue is a critical symptom commonly reported across a wide range of conditions, including oncological (24), neurological (25), and renal (26) diseases. Traditionally, the significance of fatigue in rheumatic diseases was underestimated. Nonetheless, the Outcome Measures in Rheumatology (OMERACT) conference in 2002 (27) marked a pivotal moment by formally acknowledging the importance of fatigue in musculoskeletal disorders and its profound effects on patients’ health-related quality of life. Following this, the 2007 OMERACT conference (28) recommended incorporating fatigue measurements in clinical trials for RA whenever possible. Since then, fatigue has been recognized as a key clinical symptom (29) in this inflammatory rheumatic disease and identified as a critical target for therapeutic intervention. Moreover, it is now established that fatigue stands out as a particularly significant symptom for individuals with RA (30), primarily due to its challenging management and substantial impact on all facets of daily life. Despite this recognition, there is no clear recommendation on whether to systematically analyze fatigue in clinical settings.

The estimated prevalence of fatigue in RA varies from 40 to 70% (31) and data on its prevalence in the “biological era” are limited (6, 17, 18, 32, 33). Our data, collected from elderly women with long−standing RA and assessed using the FACIT-F scale, confirm that fatigue remains a significant concern. The observed prevalence (61%) was notably high and significantly higher than in the control population (35%). Additionally, we observed that patients with RA had a mean FACIT-F value that was significantly lower than that of the control group, indicating greater fatigue severity.

The value noted in the control group (40.0 ± 9.6) was only marginally lower compared to that seen (42.7 ± 8.9) in a large sample (*n* = 1352) of the general German population (mean age: 49.9 ± 17.5 years) (34). This observation, in our opinion, underscores the appropriateness of our control group, especially in light of evidence showing that fatigue levels tend to increase with age in the general population (35).

To date, only three studies have analyzed the prevalence of fatigue in RA patients using the FACIT-F scale (17, 18, 33). Table 6 shows their main characteristics and compares their findings with those of the present study. The previous studies were cross-sectional, did not incorporate control groups, and to varying degrees, included men in their study populations.

**TABLE 6:** Characteristics of studies on fatigue in RA conducted with the FACIT-F scale.

	Pilgaard et al. (17)	Wagan et al. (18)	Kozłowska et al. (33)	Present study
Study type	Cross-sectional	Cross-sectional	Cross-sectional	Case-control
Country	Denmark	Pakistan	Poland	Spain
Number of patients	293	192	128	191
Age (years)	57.4 ± 14	39.9 ± 10.5	53.8 ± 14.5	67.5 ± 8.8
Female	79%	71,9%	85.9%	100%
Disease duration (years)	> 10 yrs: 60%	6.85 ± 4.39	11.0 ± 9.0	16.8 ± 10.3
DAS28	2.48 ± 1.11	< 3.2: 56.8%	3.8 ± 0.9	2.9 ± 1.1
FACIT-F	34.86 ± 11.04	34.94 ± 8.8	24.1 ± 9.1	36.0 ± 10.6
Prevalence of fatigue (FACIT-F < 40)	59%	62%	ND	61%

DAS28, Disease Activity Score 28; FACIT-T, Functional Assessment of Chronic Illness Therapy-Fatigue.

The obtained frequency of fatigue, and the mean FACIT-F value, in our study, was practically the same as in the studies by Pilgaard et al. (17) and Wagan et al. (18), despite differences in patient age and disease duration. In fact, as anticipated, its significantly exceeded the baseline values observed in the three clinical trials (12–14) where fatigue was assessed using this scale. The lowest FACIT-F value was observed in the study by Kozlowska et al. (33), probably because in their cohort the disease activity was clearly higher (mean DAS28: 3.8 ± 0.9).

We observed an inverse relationship between the FACIT-F scores and those of CRP, DAS28, and RAPID3. The relationship was particularly pronounced with the latter parameter, which, to our knowledge, marks the first time it has been associated with fatigue in RA patients. Interestingly, RAPID3, like FACIT-F, is a patient-reported outcome that does not require any clinical or analytical parameters and is solely based on the patient’s perception (36).

The multivariate analysis highlights RAPID 3 as a pivotal predictor of fatigue in RA patients. The significant negative relationship between RAPID 3 scores and fatigue emphasizes that higher levels of patient-reported disability and pain are strongly associated with increased fatigue. The consistent significance of RAPID 3 across various models demonstrates its robustness as a predictor, explaining a substantial portion of the variance in fatigue. This finding suggests that targeting reductions in disease activity could be a key strategy in mitigating fatigue in patients with RA.

The data obtained appear to suggest that the activity of RA exerts a deleterious effect on fatigue levels. Indeed, other researchers (4, 29) have found a positive correlation between fatigue and both the ESR and the DAS28, though not with the ratio of swollen to tender joints, with pain emerging as the predominant factor. This raises the possibility that while disease activity may be associated with fatigue, its contribution may not be significant once adjustments for pain are made.

We have not observed a correlation between disease duration and FACIT scores in our study. Research (5–7) indicates that the relationship between disease duration and fatigue in patients with RA can be variable. Some studies report that fatigue is a prevalent symptom irrespective of how long a patient has been diagnosed with RA, suggesting that fatigue can manifest in both early and long-standing cases.

As expected, we observed that patients with RA, have lower hemoglobin levels compared to the control group. However, no correlation was found between FACIT-F and this analytical parameter. This fact could suggest that hemoglobin doesn’t plays a significative role in the etiopathogenesis of fatigue, when no relevant anemia is found.

Finally, a relationship was identified between FACIT-F scores and both HAQ and SF-12, encompassing both the mental and physical components. These findings underscore the significant impact of fatigue on disability (37) and the resulting deterioration in health-related quality of life (38).

This study presents certain noteworthy limitations. Firstly, the patient cohort consisted exclusively of women. This approach, however, allowed us to focus on an often-underrepresented demographic in RA research (women with advanced age). Secondly, while the study’s single-center nature might raise questions about the generalizability of our findings, we are confident that our cohort reflects the characteristics of patients with long-standing RA typically followed in university hospitals. Thirdly, we have not systematically evaluated depression in our patients, nor have we considered the presence of this condition as an exclusion criterion. It is estimated that depression is twice as common in patients with RA as in the general population (39); additionally, previous research (40) has reported a strong association between depression and high fatigue scores. Fourthly, although we focused heavily on excluding individuals with diseases capable of inducing fatigue from the control group, the fact that some of them were selected during their hospital visits could mean that the frequency of fatigue is somewhat higher than what would have been obtained had the control group been community-based. Fifthly, given the exclusive inclusion of women in this study, our findings do not offer a comparative gender perspective on fatigue in rheumatoid arthritis; future research should aim to include a more diverse gender representation to fully explore this aspect. Sixthly, although we systematically excluded both patients and controls with conditions clearly associated with fatigue, we did not provide a detailed description of the comorbid conditions present in both groups. Seventhly, the study’s design does not allow for the establishment of causal relationships between RA characteristics and the presence of fatigue. Consequently, caution should be applied in the extrapolation of our findings. Broader, longitudinal studies encompassing more diverse populations are needed to validate and expand upon these observations.

Despite its limitations, we believe this study significantly advances the field by employing, for the first time, a case-control design to analyze fatigue frequency in a clinical setting. By using an internationally recognized and validated scale, our research not only underscores the considerable impact of fatigue on a cohort of long-standing RA patients but also reveals that fatigue prevalence significantly exceeds that of the general population. Moreover, the relationships we have identified offer interesting paths for further investigation aimed at unraveling the etiopathogenesis of fatigue in RA.

In conclusion, our findings highlight the persistent nature of fatigue in RA, demonstrating its substantial prevalence and intensity, which notably surpasses that within the general population and aligns closely with prior research, despite varying patient demographics and disease durations. Fatigue appears to be linked to disease activity, as well as with the disability and the impairment of health-related quality of life that RA entails. Given its clinical impact, in the clinical setting, it seems imperative to systematically include fatigue assessments in patient evaluations and to devise strategies aimed at minimizing the significance of this issue.

## Data availability statement

The raw data supporting the conclusions of this article will be made available by the authors, without undue reservation.

## Ethics statement

The studies involving humans were approved by the CEI IDIBELL-Bellvitge University Hospital. The studies were conducted in accordance with the local legislation and institutional requirements. The participants provided their written informed consent to participate in this study.

## Author contributions

LV-M: Conceptualization, Data curation, Investigation, Methodology, Writing−original draft, Writing−review and editing. CG-V: Conceptualization, Formal analysis, Investigation, Methodology, Supervision, Validation, Visualization, Writing−original draft, Writing−review and editing. LB-A: Investigation, Writing−original draft, Writing−review and editing. DB: Formal analysis, Investigation, Methodology, Writing−original draft, Writing−review and editing. PV-M: Investigation, Writing−original draft, Writing−review and editing. XJ: Investigation, Writing−original draft, Writing−review and editing. JN: Investigation, Writing−original draft, Writing−review and editing. JMN: Conceptualization, Formal analysis, Investigation, Methodology, Project administration, Supervision, Validation, Visualization, Writing−original draft, Writing−review and editing.
